# Development of Membrane-Based Inverted Liquid–Liquid Extraction for the Simultaneous Extraction of Eight Metals in Seawater before ICP-OES Analysis

**DOI:** 10.3390/molecules25153395

**Published:** 2020-07-27

**Authors:** Muhammad Sajid, Muhamed Kabeer, Wail Falath

**Affiliations:** 1 Center for Environment and Water, Research Institute, King Fahd University of Petroleum and Minerals, Dhahran 31261, Saudi Arabia; muhamed.korakkottil@kfupm.edu.sa (M.K.); wfallata@kfupm.edu.sa (W.F.); 2 Department of Mechanical Engineering, King Fahd University of Petroleum & Minerals, Dhahran 31261, Saudi Arabia

**Keywords:** liquid–liquid extraction, sample preparation, metal analysis, membrane-based extractions, environmental analysis, complex matrixed samples

## Abstract

In this work, we developed an extraction technique that can handle simple as well as complex matrixed liquid (aqueous) samples. In the standard liquid–liquid extraction, it is quite challenging to deal with complex liquid samples as they may complicate the process of phase separation and may lead to the formation of multiple layers. To resolve this issue, we have proposed a simple but unique idea that suggests the packing of the liquid samples inside a porous membrane bag. The edges of the membrane bag can be sealed using an electrical heat-sealer. The porous membrane bag filled with the liquid sample was immersed in an extraction solvent, and the extraction process was assisted by mechanical shaking. In order to demonstrate the proof of concept, a method was developed for the extraction of metals from seawater samples. The pH-adjusted sample, along with the complexing reagent, was packed inside the porous membrane bag, and the chelated complex was then extracted by immersing and shaking the bag inside the organic solvent. The solvent was then evaporated, and the chelated complex was dissolved/digested in acid with the aid of the heat. The final extract was subjected to Inductively Coupled Plasma-Optical Emission Spectroscopy (ICP-OES) analysis. The proposed method was used for extraction of eight metals (Cd, Co, Cu, Mo, Ni, Pb, V and Zn) from seawater samples and good extraction recoveries (75–94%) were obtained.

## 1. Introduction

Despite the considerable furtherance in analytical instrumentation, sample preparation is an indispensable step in analytical method development. Sample preparation can customarily effectuate to deal with the complex matrix or low concentrations of the analytes or analyte-instrument incompatibility. The complex matrix issue can be rectified through the cleanup of the interfering components. The enrichment of the analytes enhances the low concentrations of the analytes into a smaller volume of extraction phase relative to the volume of the original sample. The conversion of analytes into instrument-compatible derivatives resolves the analyte-instrument incompatibility complication [1,2].

Solid-phase extraction (SPE) and liquid–liquid extraction (LLE) are two commonly employed sample preparation techniques. In SPE, the analytes are separated from the sample by passing it through a solid phase, generally packed inside the column. In LLE, analytes are transferred from aqueous to organic or organic to aqueous phase by mixing the sample with an immiscible extraction phase. Despite their popularity and extensive applicability in the extraction of different classes of analytes from various matrices, SPE and LLE present several challenges concerning their classic design and dealing with complex samples [3,4]. Thus, modern extraction techniques represent improved and flexible versions of SPE and LLE that address some specific issues associated with SPE and LLE. For instance, dispersive solid-phase extraction (DSPE) is an advanced version of SPE where sorbent is dispersed inside the sample solution instead of passing the sample through a sorbent packed column. This design of SPE fully exploits the adsorption potential of the sorbent as well as resolves the issue of backpressure associated with high surface area sorbents in column format [5]. Solid-phase microextraction (SPME) is the first microextraction technique that addresses the issue of using large amounts of sorbents. Typically, SPME relies on the coating of a small amount of sorbent onto a silica fiber or metallic wire [6,7,8,9]. Similarly, liquid-phase microextraction (LPME) is a miniaturized version of LLE. It prevents the disadvantages of using high volumes of solvents and the requirement of a large volume of samples [4,10,11,12].

The search for new solutions in the area of sample preparation is a hot topic of research among analytical chemists [3]. In routine LLE, large volumes of the samples are required, and phase separation issues are prevalent while dealing with different kinds of samples. Depending on the nature of the samples, several cleanup cycles are necessary for specific situations. Multiple cleanup cycles and awkward phase separation may lead to a significant loss in the recovery of analytes. The reduction of scale and dimensions of LLE is indeed a great subject of investigation in the area of microextraction. Various new techniques, such as single-drop microextraction [13], dispersive liquid–liquid microextraction [14,15,16], and hollow-fiber liquid-phase microextraction [17,18] have been developed. The issues related to large sample volumes are resolved to a large extent, with the development of these techniques. However, the practical challenges to deal with complex samples, as well as dealing with the reduced volumes of extraction phases, limit the applications of these techniques in routine analysis. Another way to resolve the problem of the complex matrix and improper phase separation is encapsulating the liquid samples within a bag that allows the transport of the analytes but hinders the movement of complex matrix components.

The solvent-based extraction of analytes from the porous membrane packed solid samples was demonstrated for the extraction of different classes of analytes from the soil [19], tobacco [20], vegetable [21], and tea samples [22]. This approach simultaneously performs cleanup and extraction in a single step. Interestingly, it obviates the pre-extraction steps of sample treatment such as dissolution and post-extraction steps such as filtration or centrifugation. Later, another extraction technique was developed for small-volume liquid samples supported on a solid. These samples were packed into a porous membrane bag before ultrasound-assisted solvent extraction [23]. The supporting of aqueous liquid samples onto solid support like an inorganic salt may result in the dissolution of salt into the liquid sample, increasing the viscosity of the sample and, in the case of extraction of metals, ions from the salt can also interfere with the analytes. Thus, a support-less approach is required to deal with all kinds of liquid samples irrespective of their volumes.

In this work, we proposed a simple solution to deal with the problems of complex samples and issues of phase separations in LLE. The liquid sample was packed inside a porous membrane bag, which was constructed through heat-sealing. The bag was added to a solvent-containing vial. The extraction process was assisted by mechanical shaking very similar to conventional LLE. This technique was coined as membrane-based inverted liquid–liquid extraction (MILLE). The word “inverted” is used because the aqueous sample was packed inside the membrane bag, unlike other hollow-fiber- or flat-membrane-based techniques where the extraction solvent is packed inside the hollow fiber. The proof of the concept is demonstrated by the extraction of metals from seawater samples, followed by ICP-OES analysis. The final volumes usually required for inorganic analysis instruments (such as ICP-OES) are relatively high compared to organic analysis instruments. The scope of this newly developed technique is very general, and it can be used for organic analytes as well. High sensitivity in organic analysis can be achieved by reducing the volumes of the extraction solvents to desired levels, which in turn increases the enrichment of analytes.

## 2. Experimental

### 2.1. Materials, Standards, and Reagents

Polypropylene (PP) membrane sheet (Pore size: 0.1 µm, thickness: 157 µm) was obtained from Membrana (Wuppertal, Germany). An analytical standard containing a mixture of metals at concentrations of 200 mg/L and 100 mg/L was obtained from CPI International. Eight metals (Cd, Co, Cu, Mo, Ni, Pb, V, Zn) were considered in this study. The concentrations of Cd, Co, V, and Zn were 100 mg/L, while that of Cu, Mo, Pb, Ni were 200 mg/L. HPLC-grade chloroform was acquired from Fisher Chemicals (Loughborough, UK). Carlo Erba (Val de Reuil Cedex, France) supplied HPLC PLUS Gradient grade methanol and HPLC-Isocratic grade dichloromethane. HPLC-grade n-hexane was purchased from Sigma-Aldrich (St. Louis, MO, USA). Technical grade carbon tetrachloride was obtained from J.T. Baker (Phillipsburg, NJ, USA). BDH Chemicals Ltd., (Poole, England) supplied ammonium acetate and sodium hydroxide. Nitric acid was obtained from EuroStar and further purified by acid distillation unit supplied by Milestone (Headquarter located in Sorisole, Italy). Deionized water was obtained using Pure Lab Option Q provided by Elga (High Wycombe, England). Ammonium-pyrrolidin-dithiocarbamate (ammoniumtetramethylendithiocarbamate, APDC) was obtained from Fluka (Buchs, Switzerland). Diethyldithiocarbamic acid diethylammonium salt (Diethylammonium diethyldithiocarbamate, DDC) was obtained from Sigma Aldrich. The chelating reagent was a mixture of APDC and DDC, which was prepared by addition of 1 g of each in 100 mL of deionized water.

### 2.2. Preparation of Aqueous Standard Solution 

A large volume of deionized water (100 mL) was spiked with the analytical standard containing a mixture of metals to obtain final concentrations of 1 mg/L (Cd, Co, Vn, Zn) and 2 mg/L (Cu, Mo, Ni, Pb) of different metals depending on their concentrations in the original standard. For this purpose, 1 mL of the standard mixture was taken in a 100 mL volumetric flask, and the final volume was made up to mark. This aqueous solution of metals was used for the demonstration of liquid–liquid extraction and optimization of extraction parameters.

### 2.3. Membrane-Based Inverted Liquid–Liquid Extraction

**Figure 1:**
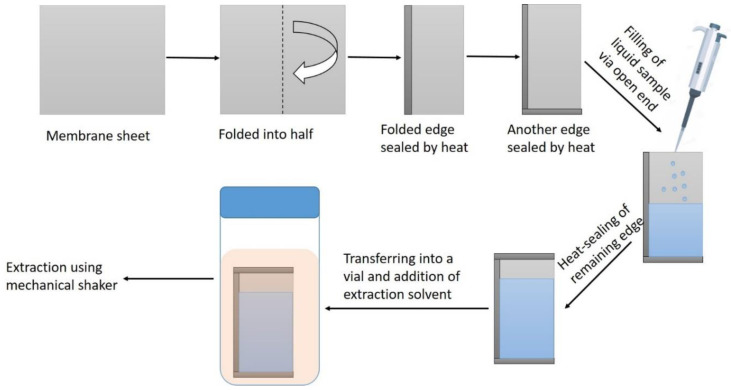
Schematic of membrane-based inverted liquid–liquid extraction (MILLE).

In the first step, the pH of the aqueous standard solution was adjusted to the desired value (pH = 4.5). Then a membrane bag was constructed through heat-sealing. For this purpose, a flat PP sheet was separated from the membrane roll. The PP sheet was folded into half, and the folded end was heat sealed. Then, another open edge was heat-sealed to shape it like an envelope with one open edge. From the open edge, 2 mL of pH-adjusted aqueous sample and 200 μL of chelating reagent were added. The open edge was then heat-sealed. Now, this was a bag filled with an aqueous sample. The sample containing bag was then placed in a 50 mL vial, and 7.5 mL of chloroform was added. Afterward, this vial was capped and placed on a shaker for 10 min for mechanical shaking and extraction of chelated metals into chloroform. The bag was then removed from the vial, and the extract was transferred into a 50 mL glass beaker. The extract was heated to dryness and then dissolved/digested using nitric acid. The final volume was then made 5 mL by using 2% nitric acid and analyzed by Inductively Coupled Plasma-Optical Emission Spectroscopy (ICP-OES) (PerkinElmer, Waltham, MA, USA). The schematic of the MILLE is shown in Figure 1.

### 2.4. Optimization of Extraction Parameters

The parameters that can potentially affect the extraction performance of MILLE were optimized. These parameters included the pH of the sample, volume of chelating reagent, type of extraction solvent, volume of extraction solvent, and extraction time. A volume of 2.0 mL of spiked deionized water samples were used for optimization studies. The recoveries of the metals were considered to select the optimum value of each parameter. The recoveries at the optimization stage were calculated using an external calibration curve built using a series of mix standards prepared in 1% ultra-pure HNO_3_ (5, 10–500 µg/L).

### 2.5. Metal Analysis by ICP-OES

The concentration of the metals was determined using ICP-OES. Table 1 lists the ICP-OES operating conditions and metals investigated in this study, along with selected wavelengths.

### 2.6. Recoveries of Analytes From Real Seawater Samples

The application of this new extraction technique was extended to real seawater samples to evaluate the effect of interfering species on the recoveries of the target metals. For this purpose, the same seawater sample was divided into six samples. Three samples were extracted as it is, while the other three were spiked. Indeed, these samples represented sample, sample duplicate, sample triplicate, spike, spike duplicate, and spike triplicate.

## 3. Results and Discussion

### 3.1. Optimization of Extraction Parameters

Some initial experiments were performed to check the extraction recoveries of different metals using MLLE. Based on encouraging values obtained for the recoveries, affecting parameters were investigated using a univariate approach.

#### 3.1.1. The Volume of a Complexing Reagent

**Figure 2:**
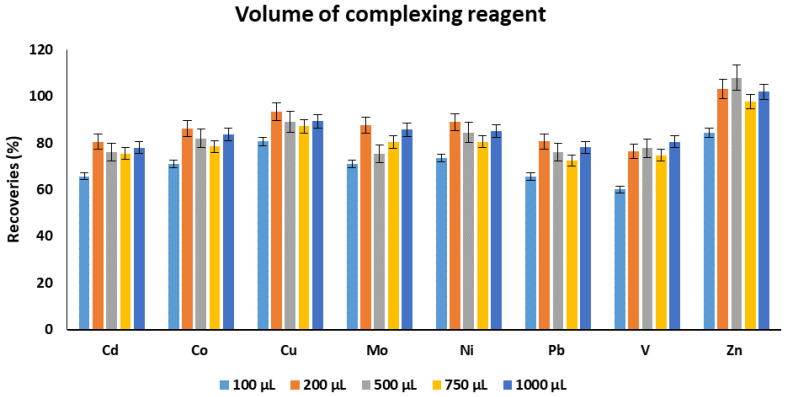
The effect of volume of chelating reagent on the recoveries of target metals. Experimental conditions: sample pH: 4.5, extraction solvent: chloroform, volume of extraction solvent: 10 mL, extraction time: 30 min.

The volume of the complexing reagent is an important parameter as it can significantly impact the recoveries of the target metals. The volume of the complexing reagent should be enough to form the complexes with available metals in the aqueous solutions. Thus, different volumes of the complexing reagent in the range of 100–1000 µL were investigated. The best extraction recoveries were achieved using 200 µL except for V and Zn. However, the difference was not very substantial. Thus, 200 µL was selected as an optimum volume for complexing reagent for subsequent experiments (Figure 2). This result indicates that 200 µL of complexing reagent is enough for the metal complexation, and any additional amount will not improve the recoveries instead a slight decrease was observed. The accurate reason for this slight decrease in recoveries is not known, but it might be due to the competitive extraction of non-chelated complexing reagent into the extraction solvent.

#### 3.1.2. The Volume of Extraction Solvent

**Figure 3:**
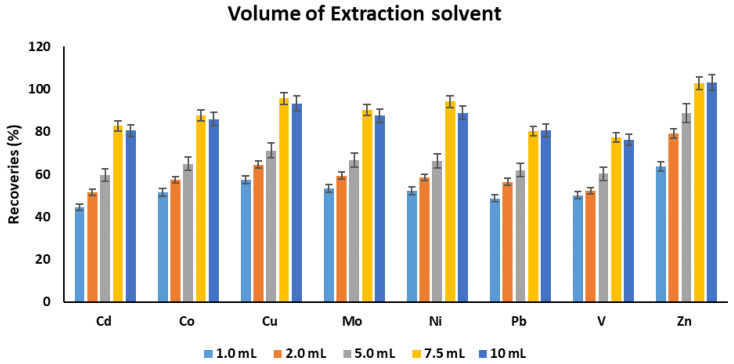
The effect of volume of extraction solvent on the recoveries of target metals. Experimental conditions: sample pH: 4.5, volume of complexing reagent: 200 µL, extraction solvent: chloroform, extraction time: 30 min.

Chloroform was initially utilized as an extraction solvent relying on our previous experience. The volume of the extraction solvent was scrutinized in the range of 1.0 to 10 mL. The analyte recoveries increased with an increase in the solvent volume from 1 to 7.5 mL, and afterward, a slight decrease was observed in cases of some elements (Figure 3). In contrast, others showed the attainment of a steady state. This indicates that volumes lesser than 7.5 mL were not sufficient to adequately extracted the target chelated metals. Hence, 7.5 mL was selected as an optimum volume of extraction solvent for the upcoming optimization experiments.

#### 3.1.3. Type of Extraction Solvent

**Figure 4:**
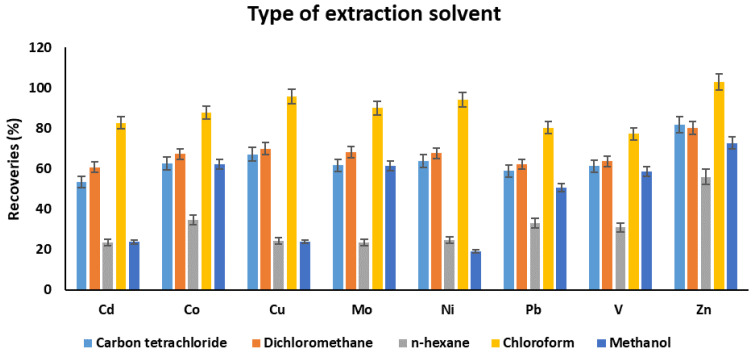
The effect of type of extraction solvent on the recoveries of target metals. Experimental conditions: sample pH: 4.5, volume of complexing reagent: 200 µL, volume of extraction solvent: 7.5 mL, extraction time: 30 min.

The selection of a suitable extraction solvent is critical to extract chelated metal complexes. In this work, different solvents, namely carbon tetrachloride, dichloromethane, n-hexane, chloroform, and methanol, were studied. As can be seen from Figure 4, the best extraction results were achieved using chloroform. The recoveries were in the following order: dichloromethane > carbon tetrachloride > methanol > *n*-hexane with some exceptions. Chloroform showed a high affinity toward chelated metal complexes compared to other solvents. Based on the highest recoveries, chloroform was selected as an optimum extraction solvent. 

#### 3.1.4. pH of Sample

**Figure 5:**
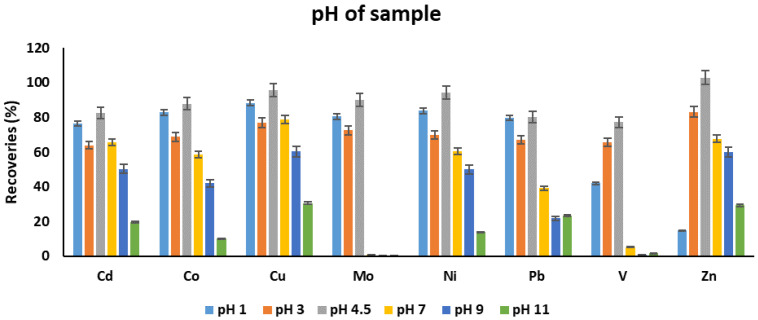
The effect of pH variation on the recoveries of target metals. Experimental conditions: volume of complexing reagent: 200 µL, volume of extraction solvent: 7.5 mL, extraction solvent: chloroform, extraction time: 30 min.

The pH of the sample solution is an important parameter for the extraction of metals because metals can exist in different forms at different pH. At the low pH values, metals usually exist in cationic form. In contrast, at higher pH values, metals do exist as their hydroxides, and precipitation may occur in the case of some metals. Apart from this, a suitable pH is the one where metal ions can form complexation with the selected chelating reagent. Thus, the analyte recoveries were determined in the pH range of 1–11. The order of recoveries was as follows: 4.5 > 1 > 2 > 7 > 9 > 11. The recoveries were increased from pH 3 to pH 4.5. After pH 7, a decreasing trend was observed (Figure 5). This may be attributed to the formation of metal hydroxides that limits the formation of metal-chelating reagent complex. Unexpected behavior was observed at pH 3. A pH value of 4.5 was found as an optimum one. The recoveries of some elements like Mo and V were substantially lost at higher pH values. 

#### 3.1.5. Extraction Time

**Figure 6:**
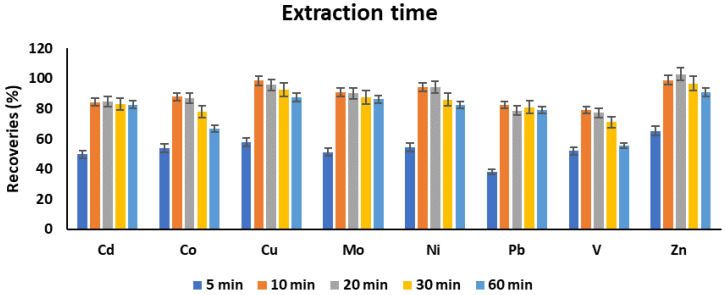
The effect of extraction time on the recoveries of target metals. Experimental conditions: sample pH: 4.5, volume of complexing reagent: 200 µL, volume of extraction solvent: 7.5 mL, extraction solvent: chloroform.

Extraction time was investigated in the range of 5–60 min. The highest analyte recoveries were obtained at 10 min extraction time, and then a steady state was achieved for the majority of the analytes except for Zn and V, for which a decrease in recoveries was recorded at 60 min (Figure 6). Although no experimental evidence can be provided, it seems extremely longer shaking times may result in slight leaching of some metals from the chelated complexes. Thus, 10 min extraction time can be selected as an optimum extraction time. 

#### 3.1.6. Salt Addition 

**Figure 7:**
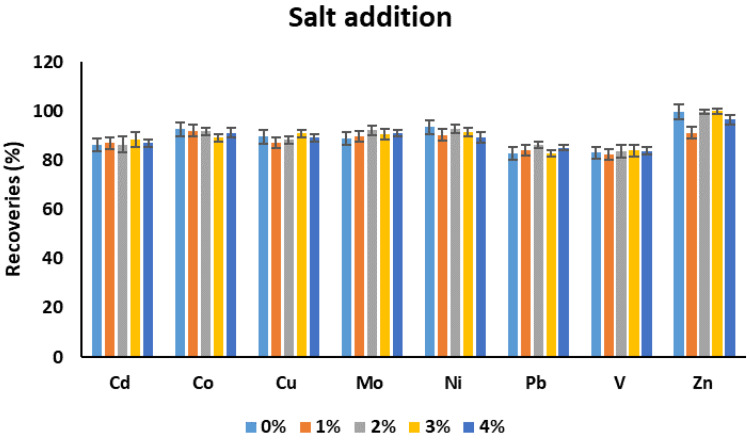
The effect of salt addition on the recoveries of target metals. Experimental conditions: sample pH: 4.5, volume of complexing reagent: 200 µL, volume of extraction solvent: 7.5 mL, extraction solvent: chloroform, extraction time: 10 min.

The effect of salt addition on the recoveries of metal was investigated (Figure 7). The amount of salt was investigated in the range of 0–4%. No significant difference was observed on the recoveries of the metal with addition of different amounts of NaCl. The salt concentration up to 4% was investigated to have a closer resemblance with the real seawater.

### 3.2. Analyte Recoveries from Real Seawater Samples under Optimum Conditions

**Figure 8:**
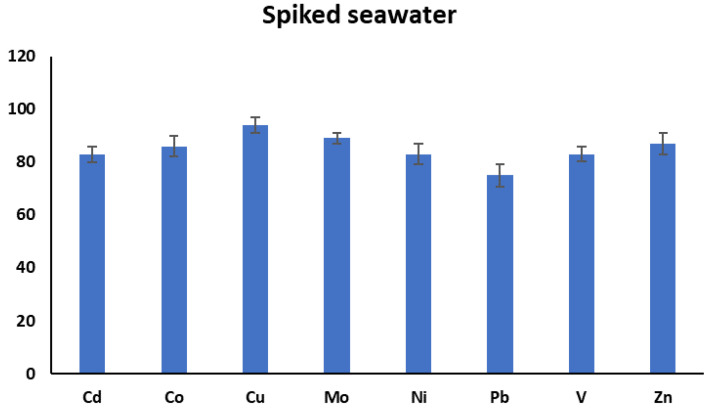
The recoveries of metals from spiked seawater samples. Experimental conditions: sample pH: 4.5, volume of complexing reagent: 200 µL, volume of extraction solvent: 7.5 mL, extraction solvent: chloroform, extraction time: 10 min.

In order to check the applicability of MILLE into the complex samples, the recoveries of analytes from spiked seawater samples were calculated. For this purpose, three unspiked and three real spiked seawater samples were extracted under the optimum extraction conditions. The mean recoveries of unspiked samples were subtracted from the mean recoveries of spiked samples. As can be seen from Figure 8, the recoveries of the spiked samples were in the range of 75–94%. This indicates that the method is equally applicable to the extraction of selected metals from seawater. The recoveries from aqueous solutions and seawater were very close to each other, implying a negligible matrix effect. 

The method’s accuracy and consistency of recoveries was further verified by spiking a certified reference material of seawater (NASS-6). The reason for spiking was the extremely low concentrations of selected elements (mostly below the instrumental and our method’s quantitation limit), in certified reference material (CRM). These concentrations can further dilute due to procedural steps. So a spiking was required to take the benefit of seawater matrix of CRM. Total concentration of each metal after spiking was considered for the calculation of recoveries. The spiked samples were extracted under optimum conditions. The recoveries from the CRM are shown in the Table 2. This confirms the consistency of recoveries from DI water, seawater, and CRM. Although the recoveries are in acceptable range, a correction factor can also be used in case of the external calibration. 

### 3.3. Analytical Figures of Merit

The calibration plots for all the analytes were also built by extracting the spiked aqueous samples under the optimum conditions. A linear response was observed in the range of 5–1000 µg/L (for Cd, Co, V), 10–1000 µg/L (for Cu, Mo, Ni, Pb, Zn). *R*^2^ values ranged from 0.9987 to 0.9999. The relative standard deviations (RSDs) were calculated at three different levels (25, 100, 500 µg/L) and were less than 6.4%. The method’s accuracy was tested by extracting drinking water CRM (1643e) and spiked seawater CRM (NASS-6). The values obtained were very close to the actual values. The results are presented in the Table 3. Three real seawater samples were analyzed using MILLE-ICP-OES and the detected concentrations are listed in Table 4.

### 3.4. Enrichment of Analytes and Final Instrumentation Technique

In order to provide the proof of concept for MILLE, 2 mL of sample volume was packed inside the porous membrane bag. The final volume of the extract was 5 mL based on the requirement of instrument-ICP-OES. In this way, a dilution of 2.5 times occurred, so no enrichment of analytes can be expected. However, the extraction performance of the technique was demonstrated by the percentage of analyte recoveries. In the case of instruments such as GC-MS, where the final required volume is few µL, the enrichment of analytes can be achieved depending on the ratio of sample volume to the final extract. This enrichment of analytes will surely enhance the method’s sensitivity and LODs. In the current scenario, to demonstrate the enrichment of metals using MILLE, we extracted 10 mL of spiked seawater sample and made a final volume of 2 mL, keeping in mind that it will lead to enrichment. The analysis was performed using ICP-OES, and only two elements Cd and Pb were considered (2 mL was not enough for eight elements using routine autosampler). In the case of Cd, an enrichment factor of 4.1 was achieved, and in the case of Pb, it was 3.8. Linear calibration graphs were obtained in the range of 1–1000 µg/L for Cd and 5–1000 µg/L for Pb with *R*^2^ values above 0.999 for both analytes using analyte-free seawater matrix. The %RSDs (*n* = 7) were determined at a spiked level of 10 µg/L and were 1.8 and 2.2 for Cd and Pb, respectively. The LODs for Cd and Pb after enrichment were 0.3 and 1.6 µg/L, respectively. The concentrations of Cd and Pb in NASS-6 (spiked at 100 and 200 µg/L) based on this calibration were found to be 98.1 ± 2.1 and 195 ± 2.6, respectively.

### 3.5. Comparison with Other Techniques

The selection of the most appropriate LLE/LPME technique depends upon

(i) The type of analyte to be determined;
(ii) The nature of the matrix;
(iii) The instrumental compatibility with the extracted phase [24].

Disadvantages of standard LLE technique are described in the introduction section. Although LPME techniques consume very little volumes of solvents, but they present several practical challenges in routine applications. Single-drop microextraction (SDME) is an attractive technique because of its simplicity and mode of operation. A single drop is used to either in headspace or direct immersion mode. The drawbacks are instability and solubility of the droplet, long extraction times. Hollow-fiber liquid-phase microextraction (HF-LPME) is good choice for complex samples because the extraction solvent is protected inside the hollow fiber. This hollow fiber leads to longer extraction times, as analytes moves through it by diffusion, the rate of analyte diffusion can be enhanced by stirring. The limitations are little volume of extraction solvent that might be difficult to retrieve from the fiber and might not suitable with the instruments like ICP-OES. Dispersive liquid–liquid microextraction (DLLME) requires very little volume and is a fast extraction technique. However, conventional DLLME requires a mixture of extraction and disperser solvents that leads to cloud formation. Extraction solvent is then separated by centrifugation. In case of low density solvents, extraction phase floats over the surface and its solidification is required before separation. The compatibility of the solvents with final instrument is also a critical factor. DLLME final extract is in microliters and it is difficult to introduce into the instruments like ICP-OES, reducing sample flow rate is an option but it may also affect the nebulization efficiency. Thus, a good extraction technique should be simple as well as operator friendly in terms of handling its different steps.

MILLE gives an opportunity to deal with complex samples and suitability of the final extract for the multielemental techniques such as ICP-OES. The required final volume can be adjusted between 2–5 mL based on the number of elements to be analyzed and this is comparable with other microextraction technique coupled with ICP-OES (Table 5). MILLE is suitable both for small- as well as large-volume samples. Here, in the optimization of MILLE, only 2.0 mL sample volume was used which is much lesser than reported in other technique. However, this volume can be varied depending upon the available sample size. The large volume samples are suitable for enrichment and sensitivity enhancement. Table 5. Compares MILLE with other techniques coupled with ICP-OES in terms of sample and final volumes (papers with different matrices are also included being few available publications on the subject).

## 4. Conclusions 

In this work, we have developed and optimized membrane-based inverted liquid–liquid extraction for extraction of metals in seawater. It is based on the packing of the liquid samples inside a porous membrane bag and then the extraction of analytes into a suitable solvent. This technique can handle both small as well as large-volume samples and presents a solution to deal with complex samples. The packing of samples inside the membrane bag results in a clean extract due to the protection of the extraction solvent from direct contact with the sample. This technique can perform simultaneous cleanup and extraction. The proof of the concept is demonstrated by the extraction of chelated metals from seawater samples followed by ICP-OES analysis. The reasonably good recoveries (75–94%) from seawater were obtained for all the tested metals. A linear response was observed in the range of 5–1000 µg/L (for Cd, Co, V), 10–1000 µg/L (for Cu, Mo, Ni, Pb, Zn). *R*^2^ values ranged from 0.9987 to 0.9999. The RSDs were less than 6.4%. The scope of this technique is extensive, and it is equally applicable to the extraction of organic analytes from liquid samples. In the case of the instruments like GC-MS, which allow having minimal final volumes, MILLE can provide very high enrichment factors and improved sensitivities of analysis.

## Figures and Tables

**Table 1:** ICP-OES operating conditions.

Parameter	Value
Plasma gas flow	10 L/min
Auxiliary gas flow	0.2 L/min
Nebulizer gas flow	0.7 L/min
RF power	1350 watts
Plasma view	Axial
Read delay	60 sec
Peristaltic pump flow rate	1.5 mL/min
Spray chamber type	Cyclonic
Nebulizer	MiraMist
Injector	Alumina
Sample tubing	Solvaflex 2-stop tubing 0.76 mm i.d.
Drain tubing	Solvaflex 2-stop tubing 1.14 mm i.d.
Torch	Quartz; single slot
Replicates	3
Wavelengths (nm)	Cd:228.802; Co:228.616; Cu:324.752; Mo:202.031; Ni:231.604; Pb:220.353; V:292.464; Zn: 213.857

**Table 2:** Analyte recoveries from spiked CRM samples under optimum extraction conditions based on an external calibration curve.

Analyte	Concentration in NASS-6 (µg/L)	Spiked Concentration (µg/L)	Total Concentration (µg/L)	Expected Concentration in Extract (µg/L) due to Procedural Dilution	Experimental Concentration in Extract, Mean ± SD (µg/L)	% Recoveries
Cd	0.0311	100	100.0	40.0	33.8 ± 0.8	85
Co	0.015	100	100.0	40.0	34.4 ± 1.4	86
Cu	0.248	200	200.2	80.1	77.3 ± 1.8	97
Mo	9.89	200	209.9	84.0	71.2 ± 0.8	85
Ni	0.301	200	200.3	80.1	72.6 ± 0.3	91
Pb	0.006	200	200.0	80.0	62.5 ± 1.8	78
V	1.46	100	101.5	40.6	36.2 ± 0.9	89
Zn	0.257	100	100.3	40.1	37.8 ± 2.1	94

**Table 3:** Analytical figures of merit of MILLE-ICP-OES (Inductively Coupled Plasma-Optical Emission Spectroscopy).

Analyte	Linear Range (µg/L)	*R* ^2^	Precision	Accuracy			
			RSDs (*n* = 7) at 25 ng/L	Actual Concentration in 1634e (µg/kg)	Concentration by MILLE-ICP-OES (µg/L)	Concentration in NASS-6 after Spiking at 50 and 100 µg/L	Concentration by MILLE-ICP-OES (µg/L)
Cd	5–1000	0.9997	3.2	6.408 ± 0.071	6.520	50.03	53.67
Co	5–1000	0.9996	4.1	26.40 ± 0.32	25.92	50.02	48.87
Cu	10–1000	0.9999	2.0	22.20 ± 0.31	22.32	100.25	99.12
Mo	10–1000	0.9996	2.8	118.5 ± 1.3	115.6	109.89	109.26
Ni	10–1000	0.9989	3.6	60.89 ± 0.67	60.95	100.30	101.34
Pb	10–1000	0.9987	6.4	19.15 ± 0.20	16.60	100.01	98.91
V	5–1000	0.9998	3.5	36.93 ± 0.57	35.56	51.46	50.03
Zn	10–1000	0.9991	5.2	76.5 ± 2.1	73.81	50.26	49.41

**Table 4:** Analysis of seawater samples using MILLE-ICP-OES.

Analyte	SW-1 (µg/L)	SW-2 (µg/L)	SW-3 (µg/L)
Cd	N.D	N.D	N.D
Co	N.D	N.D	N.D
Cu	13.5	17.1	12.2
Mo	11.9	ND	12.1
Ni	N.D	N.D	N.D
Pb	N.D	N.D	N.D
V	N.D	9.4	5.2
Zn	16.2	12.6	20.1

**Table 5:** Comparison of MILLE with other techniques coupled with ICP-OES in terms of sample and final volumes.

Extraction Technique	Sample Volume	Metals	The Volume of Extraction Solvent (Including Extraction, Disperser, Chelating Reagents) (mL)	Final Volume for Introduction to the Instrument	Real Samples	Total Sample Preparation Time (Approximate)	Recoveries (%)	Ref.
DLLME	40 mL	Cd(II), Co(II), Pb(II) and Ni(II)	8.5	2.02 mL	River water, lake water	5 min	96–106.6	[25]
HF-LPME	20 mL microwave digested sample	Ag, Al, As, Mn and Ti	20 µL of IL	5.0 mL (Phase separation by sonication and centrifugation is required)	Gasoline and diesel	112 min including microwave digestion	96–101	[26]
UA-IL-DLLME	10 mL	Sb and Sn	100–150 µL	3.0 mL	Beverages	30 min	99.2–100.4	[27]
MILLE	2.0 mL	Cd, Co, Cu, Mo, Ni, Pb, V, Zn	7.65 mL	5.0 mL	Seawater	45 min	75–94	Present work
